# A longitudinal curriculum for teaching communication skills in undergraduate dental education: Evaluation and self-assessment

**DOI:** 10.3205/zma001846

**Published:** 2026-04-15

**Authors:** Michaela Strumpski, Anja Zimmermann, Daisy Rotzoll, Rainer Haak, Felix Krause

**Affiliations:** 1 University of Leipzig, Department of Cariology, Endodontology and Periodontology, Leipzig, Germany; 2 Charité – Universitätsmedizin Berlin, corporate member of Freie Universität Berlin, Humboldt-Universität zu Berlin, Office for Study Affairs, Berlin, Germany; 3 University of Leipzig, Medical Faculty, Skills and Simulation Centre LernKlinik Leipzig, Leipzig, Germany; 4 University Hospital RWTH Aachen, Clinic for Operative Dentistry, Periodontology and Preventive Dentistry, Aachen, Germany

**Keywords:** communication, dental education, longitudinal curriculum, evaluation

## Abstract

**Purpose:**

Communicative competencies in dentistry can be taught particularly well through longitudinal curricula. The aim of this study was to evaluate a longitudinal curriculum for teaching communication skills in dental education and to examine students’ self-assessment of their communication competencies over the course of the curriculum.

**Methods:**

The curriculum was established for the 3rd to 5th year of study of dentistry. Communication skills were taught in six modules. These include theoretical units on communication and practical units, e.g., simulated patient encounters or internships for self-reflection. An evaluation accompanied the continuum of the curriculum. At four time points, students provided feedback on aspects of the curriculum. Furthermore, they assessed the development of their own communication skills via the Berlin Global Rating (BGR) and empathy via the Jefferson Scale of Physician Empathy, Student Version (JSPE-S).

**Results:**

29 students took part at all four time points T1-T4 (n=18 female, n=11 male). Most dental students felt that the curriculum was helpful or very helpful for learning patient-dentist-communication skills. Women described a significant decrease in JSPE-S-empathy over time as well as a significant increase in nonverbal and verbal expression, empathy, and coherence (BGR) over time (p<0.05). No change in communicative competencies was found in male students.

**Conclusion:**

The longitudinal, practice-oriented communication curriculum could be successfully integrated into undergraduate dental education. It fostered students’ self-confidence and, among female participants, enhanced perceived communication competencies. The results underline the value of combining simulated patient encounters, video-based peer feedback, and guided self-reflection in preparing dental students for effective patient-centered communication.

## Zusammenfassung

**Ziel:**

Kommunikative Kompetenzen in der Zahnmedizin sind durch longitudinal angelegte Curricula gut vermittelbar. Ziel dieser Studie war es, ein longitudinales Curriculum für die Vermittlung kommunikativer Kompetenzen in der zahnmedizinischen Ausbildung zu evaluieren und die Selbsteinschätzung der Studierenden hinsichtlich ihrer kommunikativen Fähigkeiten im Verlauf des Curriculums zu untersuchen.

**Methoden:**

Das Curriculum wurde im dritten bis fünften Studienjahr in die zahnmedizinische Ausbildung implementiert. Die kommunikativen Kompetenzen wurden in sechs Teilen vermittelt. Dazu gehörten theoretische Einheiten zum Thema Kommunikation sowie praktische Einheiten, wie simulierte Patient*innengespräche oder Übungen zur Selbstreflexion. Der Verlauf des Curriculums wurde durch eine Evaluation begleitet. Zu vier Zeitpunkten gaben die Studierenden Feedback zu verschiedenen Aspekten des Curriculums. Darüber hinaus bewerteten sie die Entwicklung ihrer eigenen Kommunikationsfähigkeiten anhand des Berliner Global Ratings (BGR) sowie ihrer Empathie mit der Studierendenversion der Jefferson Scale of Physician Empathy (JSPE-S).

**Ergebnisse:**

29 Studierende nahmen an allen vier Zeitpunkten T1-T4 teil (n=18 weiblich, n=11 männlich). Die meisten Zahnmedizinstudierenden empfanden das Curriculum als hilfreich oder sehr hilfreich für das Erlernen kommunikativer Kompetenzen im Umgang mit Patient*innen. Frauen beschrieben einen signifikanten Rückgang der JSPE-S-Empathie im Laufe der Zeit sowie einen signifikanten Anstieg der nonverbalen und verbalen Ausdrucksfähigkeit, von Empathie und Kohärenz (BGR) im Laufe der Zeit (p<0,05). Bei den männlichen Studierenden wurde keine Veränderung der Kommunikationskompetenzen festgestellt.

**Schlussfolgerung:**

Das longitudinal angelegte, praxisorientierte Kommunikationscurriculum konnte erfolgreich in die zahnmedizinische Ausbildung integriert werden. Es förderte das Selbstvertrauen der Studierenden und verbesserte die wahrgenommene Kommunikationskompetenzen der weiblichen Teilnehmerinnen. Die Ergebnisse unterstreichen den Wert der Kombination aus simulierten Patientengesprächen, videobasiertem Peer-Feedback und angeleiteter Selbstreflexion bei der Vorbereitung von Zahnmedizinstudierenden auf eine effektive patientenzentrierte Kommunikation.

## 1. Introduction

Communication skills and the establishment of a trustful dentist–patient relationship are essential competencies for every dentist [1], [2]. Professional communication enables accurate identification of patients’ problems, increases patient satisfaction, reduces anxiety, and decreases work-related stress for the dentist [3], [4], [5].

It is well established that communication skills can be taught and learned, and several studies have examined how this learning effect can be measured [6]. The importance of teaching communicative abilities in dental education has been emphasized repeatedly [7], [8]. Consequently, communication skills courses have become integral components of dental curricula in many countries [9], [10], [11], [12], [13]. Van Dalen, for example, demonstrated in a comparative study that integrated longitudinal curricula are significantly more effective than isolated preclinical courses. In line with this, several consensus statements recommend longitudinally integrated communication skills training rather than stand-alone interventions [14], [15], [16].

Empirical evidence has identified seven key methods as particularly effective for teaching communication skills in medical and dental education [17], [18], [19], [20]. Each of these methods contributes distinct advantages to the learning process:

1. Skills-based approach – emphasizes the structured acquisition and repeated practice of specific techniques such as questioning, summarizing, and active listening. Studies demonstrate that such structured training leads to measurable and sustainable improvements in communication performance [17].
2. Clinically relevant scenarios – provide realistic and context-based learning opportunities that enhance motivation and facilitate the transfer of skills to clinical practice. Evidence shows higher engagement and better long-term outcomes compared to abstract exercises [18].
3. Self-assessment by students – fosters reflection, metacognitive awareness, and personal responsibility for improvement. Research indicates a positive correlation between self-assessment and subsequent performance in communication tasks [19].
4. Videotaping methods – enable detailed observation and analysis of student–patient interactions, promoting self-reflection and targeted feedback. Numerous studies report significant gains in communication competence, self-awareness, and empathy through video-based learning [20].
5. Simulated patients with feedback expertise – are widely recognized as one of the most effective approaches. Trained standardized patients provide structured feedback from both patient and observer perspectives, enhancing authenticity, emotional engagement, and the development of empathy and professionalism [18], [20].
6. Integrated teaching teams – involving clinicians, psychologists, and communication experts combine clinical authenticity with behavioral and social science perspectives. Interdisciplinary teaching formats are highly valued for their comprehensiveness and transferability to real-life practice [19].
7. Small-group learning – promotes active participation, peer feedback, and experiential learning in a supportive environment. Studies confirm that small-group settings lead to greater satisfaction and improved communication performance compared with large lectures [17], [18].

Overall, these methods are consistently rated as effective and complementary, with the strongest evidence for interactive, feedback-oriented approaches such as simulated patient encounters and video-based reflection. In contrast, passive instructional methods (e.g., lectures, presentations) are mainly valuable for theoretical preparation and conceptual understanding [21], [22], [23]. Dentists must not only understand communication theory but also demonstrate the ability to ask appropriate questions, listen actively, and communicate clearly and empathetically [24].

An essential goal of communication curricula is therefore to motivate students to interact with patients in an appreciative, empathic, and patient-centered manner – strengthening the dentist-patient relationship and promoting patient compliance. Taking the patient’s perspective helps students understand patients’ thoughts, emotions, and expectations [25]. However, research on the development of empathy among medical and dental students shows inconsistent findings: while some studies report an increase in empathy during training, others describe stagnation or even decline [26], [27], [28], [29], [30]. When looking at gender differences in empathy, studies found significantly higher empathy scores among female medical and dental students [31], [32].

Since empathic behavior can be taught and learned and constitutes a fundamental element of professional communication, it should be deliberately integrated into a longitudinal curriculum [33].

To ensure credibility and effective transfer of learning, communication courses should include practical elements that are directly related to dental practice. Khalifah et al. [34] identified four major categories of communication skills relevant to dental education:

1. generic skills (e.g., active listening, empathy, nonverbal communication),
2. case-specific skills (e.g., structuring consultations),
3. time-specific skills (e.g., initiating or closing sessions), and
4. emerging skills (e.g., cultural sensitivity, obtaining consent). Commonly used teaching strategies include lectures, workshops, role-play, simulated patients, and video-based exercises [35].

Among all instructional components, feedback is recognized as a key determinant of learning success. It promotes self-awareness, autonomy, and measurable improvement in both self-perceived and objectively assessed communication competence [36]. Particularly video feedback, based on recorded encounters followed by guided reflection with peers or experts, has proven highly effective in fostering communication skills and empathic understanding [37]. Interestingly, the communication experience of the feedback provider appears to have little influence on learning outcomes regarding empathy, coherence, or nonverbal expression [38]. Students consistently report that videotaping and peer-group feedback provide some of the most beneficial learning experiences for improving their communication skills [39].

Despite the availability of various approaches, a comprehensive longitudinal communication curriculum for undergraduate dental students that combines practical elements, video-based peer feedback, and systematic self-reflection has so far been lacking. The present study was therefore designed to fill this gap. A longitudinal communication curriculum for dental students at a German medical faculty was implemented and evaluated, employing multiple evidence-based teaching methods to support differentiated and sustained learning.

The aim of this study was to evaluate the contents of a longitudinal communication curriculum in undergraduate dental education and to analyze the development of empathy and self-perceived communication skills over time and in terms of gender differences throughout the program. It was hypothesized that undergraduate dental students would perceive an improvement in their communicative competencies and show an increase in empathy during the communication curriculum. It was also assumed that female dental students show higher levels of empathy than their male peers.

## 2. Methods

### 2.1. Study design and participants

The current study was a cohort study with prospective follow-up over three academic years. The study was approved by the Ethics Committee of the Medical Faculty of Leipzig University, Germany (No. 378/15-05102015). All students were informed verbally and in writing and gave their written consent for participation.

### 2.2. Study flow

**Figure 1:**
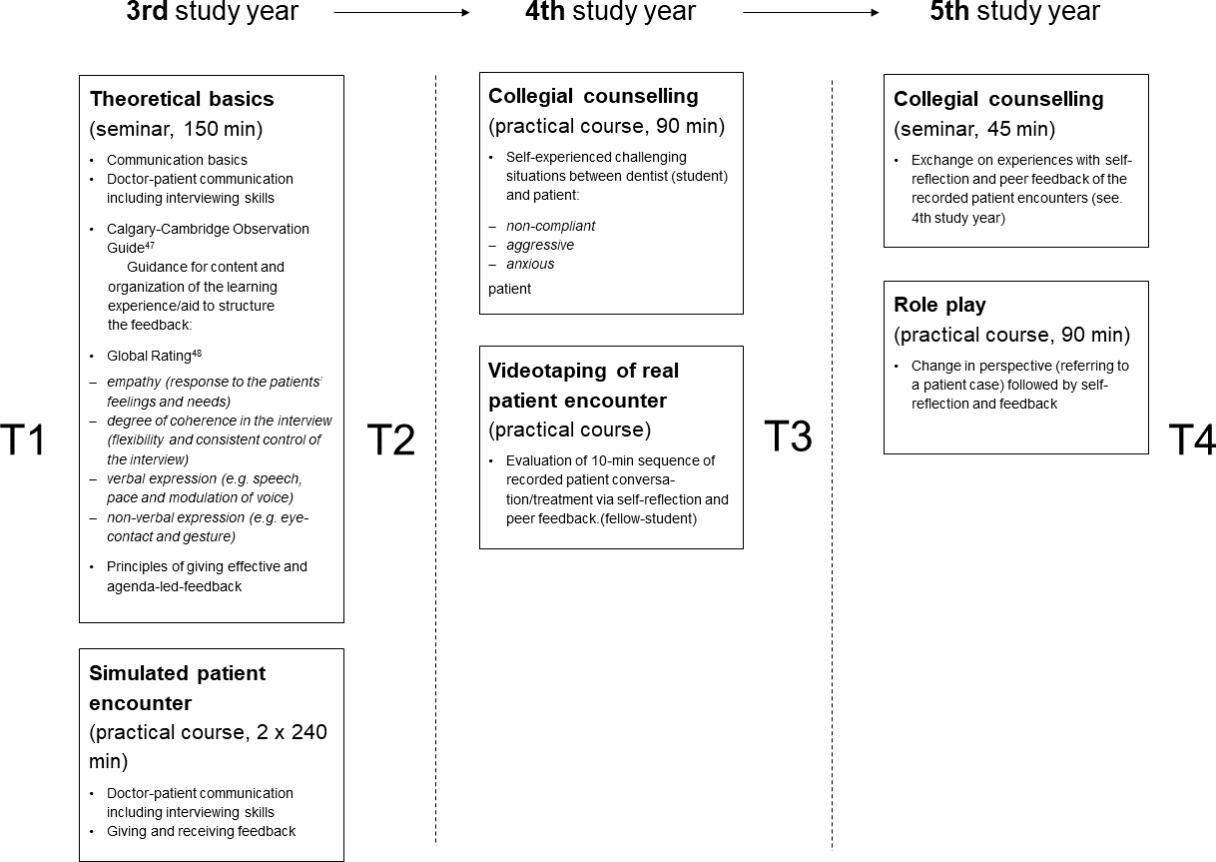
Design and content of the curriculum with assessment time points (T1-T4)

Following pilot projects [38], [39], the longitudinal communication curriculum was implemented in the clinical years from the 3^rd^ to 5^th^ study year. The curriculum consisted of six parts, each year comprising two parts (see figure 1 (Fig. 1)).

### 2.3. Communications content of the curriculum

#### 2.3.1. 3^rd^ study year

The longitudinal curriculum started with a 150-minute introductory seminar that addressed the following communication basics: oral and written communication, communication models [40], [41], four sides of a message [42], [43]; active listening and nondirective interviewing [44], [45], [46], [47], questioning and interview techniques.

Furthermore, students were introduced to the Calgary-Cambridge Observation Guide (CCOG), which delineates and structures doctor-patient communication [48], and they learned about the Berlin Global Rating Scale (BGR) as an instrument for analysing communication [49], [50]. Both instruments, the CCOG and the BGR, were used to analyse the patient-doctor interview. Furthermore, the principles of giving and receiving structured feedback were discussed, and communication principles in medicine and dentistry were demonstrated with the help of video examples. They were then discussed with regard to alternative ways of behaviour. All the theoretical content presented was made available in writing in a compendium for the students’ self-study. 

In two practical sessions of 240 minutes each, each student conducted a patient interview with a simulated patient (SP). The 4 roles and up to 2 situations per role for the simulation were developed in an interprofessional team consisting of a psychologist (AZ), and two dentists (MS, FK) experienced in communication. The SP encounters were conducted in small groups of about 6-7 students. While one student conducted the conversation with the SP, the other students observed the video-transmitted conversation in another room. Afterwards, the student conducting the conversation was asked to reflect on the conversation itself. Afterwards, he received structured feedback from the SP, his fellow students and the psychologist or dentist. Thus, each student experienced one SP encounter including self-reflection and for which they received a structured feedback. Each student observed a total of six other SP encounters with fellow students, on whose conversations they were asked to give structured feedback.

#### 2.3.2. 4^th^ study year

In the 4^th^ year of study, dealing with self-experienced challenging situations between dentist (student) and patient was addressed in a 90-minute seminar with the help of collegial counselling. The content was about complex consultations, e.g. non-compliant, aggressive or anxious patients. The students took on the roles of moderator, facilitator and observer.

Furthermore, each student videotaped one of their own real patient encounters. The subsequent evaluation of the recorded patient conversation/treatment was performed through self-reflection and peer feedback. For this purpose, the students selected a 10-minute section of their videotaped examination, in which they reflected on themselves and received feedback from a fellow student. The feedback was given both orally and in writing. The corresponding procedure is described in Krause et al. [38].

#### 2.3.3. 5^th^ study year

In the 5^th^ year of study, the experiences with self-reflection and peer-feedback of the recorded patient conversations were discussed in a 45-minute seminar. For this purpose, the students were given the opportunity to talk about their experiences as well as problems they encountered. In another 90-minute seminar, change of perspective was analyzed. In two groups, the students worked on a challenging patient case. This included both the reason for the encounter and the patient’s character. In a second step, the patient was played by a student from the respective group. A student from the second group took on the dentist's role who had to take the medical history. Afterwards, there was feedback and self-reflection from the perspective of both the dentist and the patient. The roles were then switched, with the first group taking on the dentist’s role and the second group taking on the role of the patient.

### 2.4. Evaluation questionnaires

#### 2.4.1. Assessment points for evaluation

The evaluation was conducted at four points in time (T1-T4, see figure 1 (Fig. 1)). Students were asked to fill out the questionnaires before starting the longitudinal curriculum (T1), after the SP encounters (T2), after the evaluation of the videotaped patient interviews (T3) and at the end of the curriculum (T4). The survey was anonymous. In order to be able to evaluate the data longitudinally, the students generated a personal code to allow intraindividual data comparison. The survey was carried out on paper to increase the response rate.

#### 2.4.2. Evaluation instruments

The Jefferson Scale of Physician Empathy, Student Version (JSPE-S) was used to measure the relevance of empathy in the dentist-patient relationship [51]. The JSPE-S is widely used to measure physician empathy in medical students [26], [29], [51] and is equally well known as an instrument for the reliable and valid measurement of empathy in dental students [27]. Students answer 20 items on a 7-point Likert scale (1=strongly disagree – 7=strongly agree). The total score ranges from 20 to 140, with a higher score indicating greater empathy. In the present sample, Cronbach’s alpha was 0.70, lower than in* another* German sample of medical students with 0.83, but still acceptable [52]. The JSPE-S was used at T1, T3 and T4.

The German version of the Berlin Global Rating Scale (BGR) was used to measure communication skills [53]. The scale consists of 4 subcategories, each representing one dimension: empathy, structural coherence, verbal and non-verbal expression. Key statements were used to assess each dimension. The BGR consists of a 5-point Likert scale: “1” is the most positive and “5” is the most negative value. The BGR was used for students’ self-assessment of communication skills. This scale has been used before in a similar way [38] for peer and tutor assessment, whereas this study used self-assessment at all assessment time points (T1-T4).

#### 2.4.3. Curriculum evaluation items

The curriculum, in general, was evaluated by a self-constructed questionnaire using a 5- or 6-point Likert scale. Students rated different aspects of the curriculum according to how helpful they were or how much an aspect contributed to improving their own communication skills at T2, T3 and T4. For this purpose, both the SP encounters and the practical courses were evaluated (see table 1 (Tab. 1)). The 5-point Likert scales were used when students were asked to assess their own learning progress, with 3 being given as a “neither - nor” option. A 6-point Likert scale was used for assessments of the importance of communication in dentistry. The 6-point scale was intended to avoid a tendency toward the middle.

### 2.5. Statistical analysis

SPSS^®^ 25 for Windows was used for data analysis. The evaluation results and changes over time are presented as frequencies, means and standard deviations. A p-value of <0.05 was regarded as statistically significant. ANOVA eta^2^ [54] was used to interpret the effect size.

Due to the small sample size, differences at T1 were controlled by using the Mann-Witney U test. For the evaluation of the curriculum, individual items were analyzed. Two groups with regard to gender were defined to analyse differences.

It was expected that self-assessed communication skills would improve over time. Furthermore, it was assumed that an increase in empathy could be observed. In order to check whether there was a gender effect, gender was included as a variable in the calculation.

For the analysis of this changes over time with regard to gender for empathy and communication skills, two-factor ANOVA with repeated measurements was carried out. 

Despite the small sample size, an ANOVA was used to test both the change over time and the difference between genders simultaneously. As this is a robust method, it was decided to use this approach despite the small sample size. To further validate the results, a Friedman analysis was additionally performed to show differences between the three measurement time points. These analyses were conducted separately depending on gender.

## 3. Results

### 3.1. Participants

At the baseline (time point T1), 40 third-year dental students (n=27 female, n=13 male) with a mean age of 22 years (20-35 years), participated in the study. Five of them stated that they already had medical experience. Of all students, N=31 (78%) were re-evaluated at T4. Due to the COVID-19 pandemic, only half of the cohort was able to participate in the unit “role play”. Nevertheless, the entire cohort was asked to complete the evaluation. The changes over time were evaluated up to T3 so that the results were not distorted by this difference in the number of participants. For completeness, the evaluation results at T4 are presented. However, only the frequency is reported here. Therefore, the results at T4 should be interpreted with caution, as half of the students only participated in two teaching units. These were asked to evaluate the units they participated in.

Not all participants could be matched to their personal code number; at the end, a total of n=29 (=73%) participants filled out questionnaires at T1, T2, T3 and T4. The sample included 18 female and 11 male students with a mean age of 23 years (median 22, range 20-30).

At T1, there were no gender differences for the global BGR scales. However, a difference was found for JSPE-S empathy. Women rated the relevance of empathy higher than men (U=52.5, p=0.035).

### 3.2. Longitudinal curriculum content evaluation

#### 3.2.1 Assessment point: T2

At T2, students were asked what communication in the dentist-patient relationship means to them, with 1 being the most positive answer and six the most negative. The content of dentist-patient communication in dental education is seen as positive overall (mean=1.66, SD=0.86). Students consider that they can improve their communication skills themselves (mean=1.86, SD=0.95) and believe that communication skills can be learned at all (mean=2.00, SD=0.80). The students rate dentist-patient communication as a core dental skill (mean=1.83, SD=0.89). At T2, the communication curriculum was rated as “good” (school grade, mean=1.85, SD=0.82).

#### 3.2.2. Assessment point: T4

**Figure 2:**
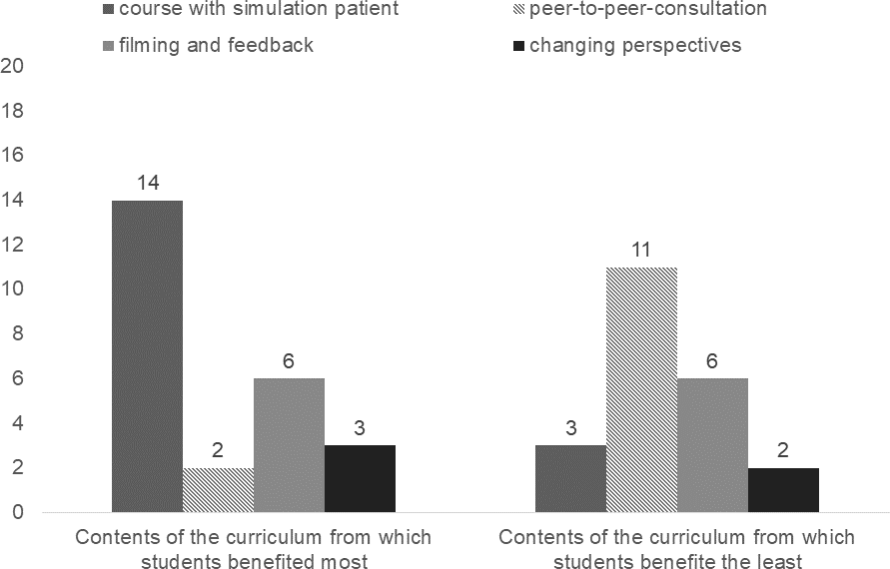
Students’ rating of which content of the curriculum they benefitted from most/least T4, n=31, multiple answers possible

Students at T4 stated that they benefitted the most from the SP encounter (n=14), whereas the peer-to-peer consultation course was the one students stated that they had benefitted the least (n=11). Videotaping one’s own treatment with subsequent feedback and the change of perspective were rated contradictorily (see figure 2 (Fig. 2)).

The SP encounter was seen as helpful in learning patient-dentist communication (86% helpful and very helpful). 

After the peer-to-peer consultation, the curriculum was rated as helpful for learning patient-dentist communication by 62%; 73% found the curriculum helpful or very helpful at the last survey time point T4.

#### 3.2.3. All assessment points

**Figure 3:**
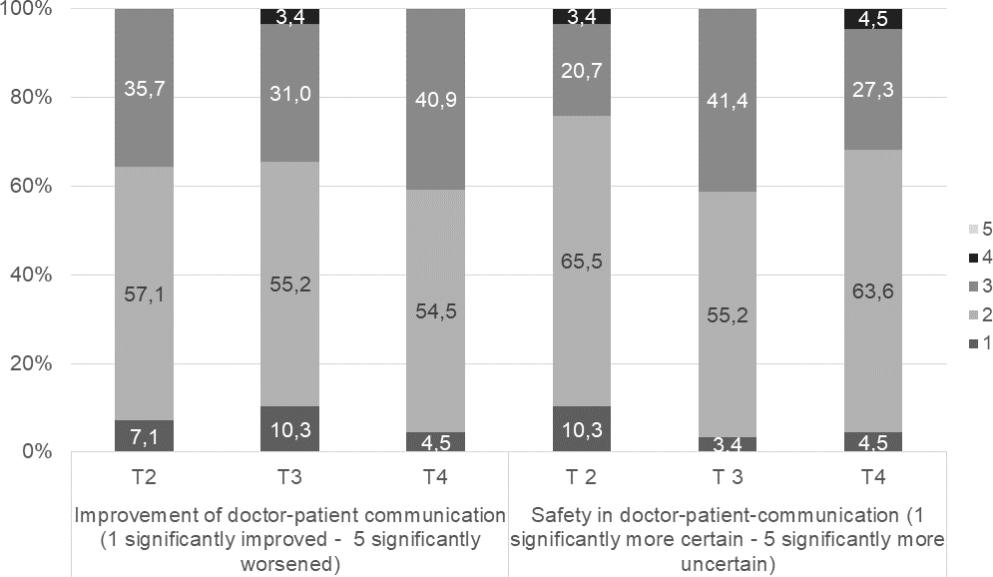
Student evaluation of communicative skills (self-perceived improvement and confidence) over time T2 and T3 n=29, T4 n=31

Most dental students reported both a subjective improvement and greater confidence in their communication skills at all survey time points (see figure 3 (Fig. 3)).

### 3.3. JSPE and BGR changes over time depending on gender

Students described a significant decrease in JSPE-S from T1 to T3, with a strong main effect (eta^2^=0.26). Other main effects in their self-assessment could be found for coherence and non-verbal expression of BGR. For coherence, there was a medium main effect of time (eta^2^=0.12). Students described a significant increase in coherence from T1 to T2 (p=0.013) and from T1 to T3 (p=0.027). No difference was found between T2 and T3 (p=0.965).

For non-verbal expression, students described a similar change. The main effect for non-verbal expression was strong (eta^2^=0.19, [55]: students described an increase from T1 to T2 (p=0.003) and from T1 to T3 (p=0.024). No statistical differences were found between T2 and T3 (p=0.409; table 2 (Tab. 2) for the complete results).

No main effects for time or gender were found for the self-assessment for the empathy scale and the verbal expression of BGR (see table 2 (Tab. 2)). For the empathy scale, there was a correlation between time and gender. While women described that their empathic skills improved from T1 to T3, men described a decrease. This was a strong effect (eta^2^=0.25). The same was found for the verbal expression scale with a strong effect (eta^2^=0.30). Women described an improvement; men experienced the opposite development (see table 2 (Tab. 2)). 

Due to the small sample, a Friedman ANOVA was additionally conducted. Self-reported improvements in communication skills where found in women in the BGR scales empathy (women χ^2^=9.60, p 0.008), coherence (women χ^2^=11.88, p=0.003), verbal (women χ^2^=19.85, p<0.001) and non-verbal expression (women χ^2^=10.43, p=0.005). Women described a decrease in JSPE empathy over time (women χ^2^=5.56, p=0.018). No significant changes were found in men, except that there was a tendency for BGR empathy in men to decrease in their self-assessment (men χ^2^=5.56, p=0.062).

## 4. Discussion

This study shows that most dental students reported improvements in their communication skills during the longitudinal communication curriculum for undergraduate dentistry students described here. They felt that the SP encounter helped to learn patient-doctor communication. This corresponds with a systematic review that showed that dental students find SP encounters beneficial [7]. The curriculum is evaluated as helpful by a majority of dental students at every measurement point. They felt they could improve in communication skills and even felt that dentist-patient communication is a core competency. The positive attitudes towards learning communication skills in a longitudinal curriculum are consistent with a previous dental study [56]. It can be stated that the curriculum meets the needs of dental students at Leipzig University. In the 4th and 5th study year units, improvements can be made. Students shall be allowed to learn even more in a practical manner. Other studies show that explaining specific skills in a practical dental situation will make the necessity of mastering these skills much more visible [9], [10]. For sure, it must be taken into consideration that training communication skills is just one part of dental studies, students have limited capacity, and it has to fit in the curriculum. On the other hand, it is desired by students that communication competencies are included in dental education [39].

In the curriculum, self-evaluation was used to assess the own communicative competencies. Self-evaluation develops problem-solving, autonomy and critical thinking. The reflective student creates meaning from events that have transpired to guide future choices [55]. Another approach could be to assess communicative skills by peers. Research shows that students received significantly more positive comments from their peers than from themselves. Students were also ranked higher by their peers than by themselves [57]. Regardless of the form that in-process formative assessment takes, it should seek to optimise learners’ skills by guiding future learning of communicative skills.

At the beginning of the curriculum, women judged the relevance of empathy higher compared to men. In progress relevance of empathy decreased, especially in women, as Friedman-Anova showed. Men reported no change. Possibly women start at a higher level than men and then converge to the same level. This decrease of empathy has been found before in dental [27] and medical students [29]. For this, it is not surprising that similar results were found. One explanation of the decrease is the student exposure to more clinical skills [30] or a high pressure due to large learning loads or time limits [26].

The Berlin Global Rating Scale (BGR) was developed as an observation tool for external assessment of communication skills in medical consultation situations [50], [53]. In our study, it was also used for self-assessment by students. Accordingly, there are limitations regarding the validity and reliability of the self-assessments. In addition, the use of the scale requires a degree of analytical distance that is only available to a limited extent in self-observation. Comparable rating instruments also show that external raters can make finer distinctions than the person concerned [58]. However, the structured orientation of the BGR provides learners with guidance for reflecting on their own conversational behaviour. Integration into training concepts that combine self-assessment and external assessment can increase the learning value of the scale and contribute to the development of communication skills. Furthermore, comparing self-assessments and external assessments can help raise awareness of discrepancies between self-perception and external perception, which is described in the literature as conducive to learning [59]. Examination of real patients and high work load prior to examination explain the decreasing perception of the relevance of empathy as measured with JSPE. The relevance of empathy in medical and dental students also shows differing results in the literature. Some studies show an increase in self-perceived relevance, whereas others show a decreased or stable self-reported perception of the relevance of empathy [26], [27], [28], [29], [30]. However, the decrease of the relevance of empathy female students report an increase in the target variables empathy, nonverbal and verbal expression and coherence. While relevance decreases over time, the self-perceived ability to act empathetically increases. Interestingly, this can be seen for women but not for men. Men did not profit the same way. They even tend to decrease in empathy. These results are in line with research on gender differences in empathy: men and women have been found to have different empathetic traits [60], [61], [62], and training courses are more effective for women than men [63]. Maybe the men of this group judged themselves higher in communication skills per se, so they cannot increase the way women do. The fact that there was no significant difference in communication skills between men and women before the course argues against this. Maybe women take the chance to exercise and self-reflect more than men do. There is also the possibility that a communication course and a repeated reflection about own communication skills help correct self-perception. Men may overestimate their communication skills and adapt their self-assessment over time, while women experience an amelioration over time. 

### 4.1. Strengths and limitations

The communication curriculum could be established at Leipzig University. It is, as far as we know, the first longitudinal, integrated communication curriculum for undergraduate dental students in German-speaking countries (Germany, Austria, Switzerland) that includes the work with simulated patients in clinically relevant scenarios, video-based peer-feedback and self-perception and thus meets the needs of the students [39]. It was developed and carried out by an interprofessional team of a psychologist and dentists, thus combining communicative expertise with the dental practice. The curriculum was evaluated at different time points, so an apparent strength is that we have data from 29 students over two years of learning communicative competencies.

One study year has gone through the adapted curriculum. Due to the COVID-19 pandemic, the second and third cohorts could not accomplish the curriculum as planned. Therefore, several adapted online courses were conducted. Despite this, the longitudinal communication curriculum is now a part of dental studies at Leipzig University.

Only self-assessment instruments were used; accordingly, the development over time is very subjective. The sample size is very small and even smaller for men than for women. The results should be interpreted cautiously and should be further investigated on a larger sample if possible. A multicentre study with other medical faculties would be desirable. No control group was included, so it remains questionable whether changes are natural or caused by the communication skills training. On the other hand, it would be difficult to justify not offering students opportunities to acquire communicative skills. In the future, control groups that enter the training later could be used to study what effect the communication courses have on the students compared to the “normal” course of study.

In a follow-up study, self-report and extern objective measures should assess communication skills and the relevance of empathy. A reproducible outcome criterion could be collected with an OSCE test for communication skills, whereby the complexity of the different scenarios strongly influences the validity concerning communication skills in everyday medical practice. In the future, it will be necessary to determine to what extent self-assessed and "objectively" observed communication skills correspond or differ. Another SP encounter at the end of the curriculum could be highly beneficial for dental students. However, there is currently a lack of financial capacity locally, so future curriculum development should take these resources into account to establish the highest quality feedback for students.

## 5. Conclusion

This study demonstrates that a longitudinal, practice-oriented communication curriculum can be successfully integrated into undergraduate dental education. It fostered students’ self-confidence and, among female participants, enhanced perceived communication competencies. The results underline the value of combining simulated patient encounters, video-based peer feedback, and guided self-reflection in preparing dental students for effective patient-centered communication. Further multicenter studies with larger cohorts and objective assessments are warranted to consolidate these findings and guide future curriculum development.

## Acknowledgements

We would like to thank all dentistry students who participated in this study and provided valuable feedback for further development. 

## Abbreviations

- BGR: Berlin Global Rating
- CCOG: Calgary-Cambridge Observation Guide
- JSPE: Jefferson Scale of Empathy
- SP: Simulated patient
- T1-T4: Assessment time points

## Notes

### Availability of data and materials

The datasets used and analysed during the current study are available from the corresponding author on reasonable request.

### Ethics approval and consent to participate

The project was approved by the Ethics committee of the Medical Faculty of the University of Leipzig, Germany (No. 378/15-05102015). 

All students provided written informed consent to participate in the study.

Written informed consent was obtained from every patient (and in the case of children from their parents) to use the videotapes for teaching and study purposes. 

### Authorship

Michaela Strumpski and Anja Zimmermann contributed equally as first authors to this work.

### Authors’ contributions

- MS made substantial contribution to the development and design of the study, interpreted the data and was a major contributor in writing the manuscript.
- AZ had a substantial contribution to the development and design of the study, analysed and interpreted the data and was a major contributor in writing the manuscript.
- RH and DR have substantively revised the work and contributed to the conception and design of the study.
- FK had a substantial contribution to the conception and the design of the study and substantively revised the work.

All authors read and approved the manuscript.

### Authors’ ORCIDs

- Michaela Strumpski: [0009-0000-8397-8941]
- Daisy Rotzoll: [0000-0002-0087-8249]
- Rainer Haak: [0000-0002-3716-4231]
- Felix Krause: [0000-0001-5732-5752]

## Competing interests

The authors declare that they have no competing interests. 

## Figures and Tables

**Table 1:**
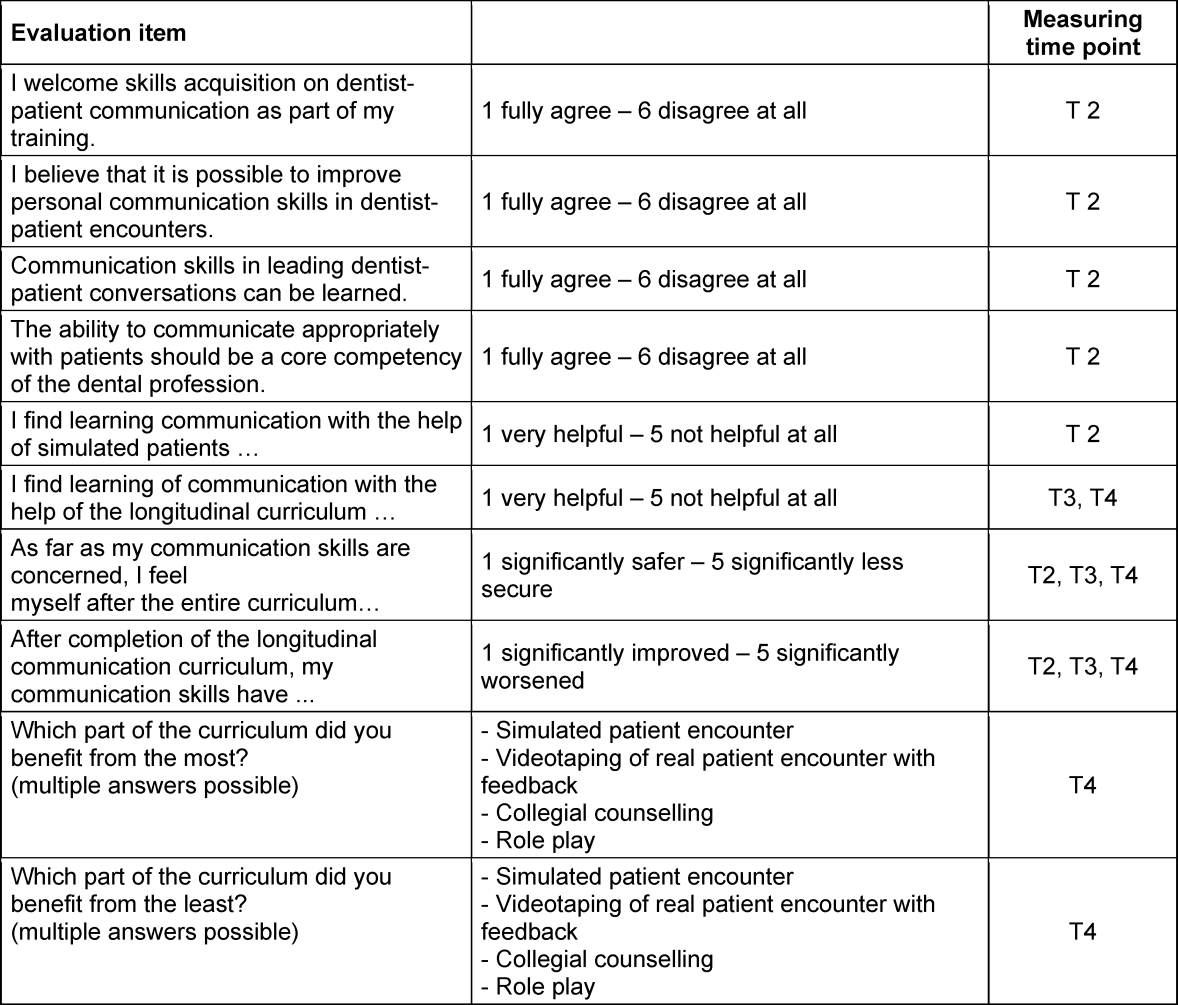
Evaluation items at different measuring time points

**Table 2:**
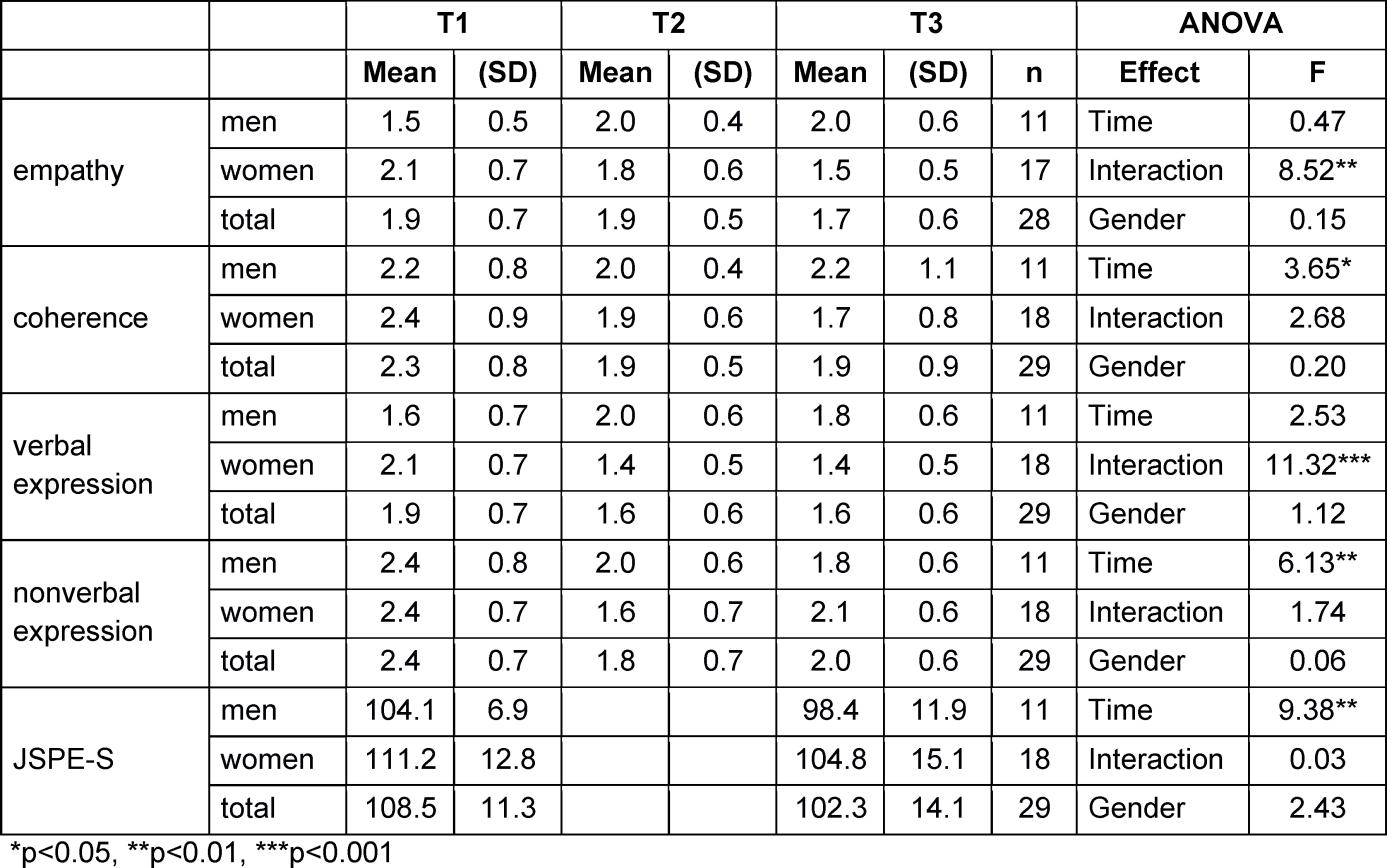
Changes for BGR and JSPE-S, two-factor-ANOVA with repeated measures considering time and gender
